# Antipsychotic and Psychopharmacological Medications: Care Transitions Among Nursing Home Residents

**DOI:** 10.1093/geroni/igab046.345

**Published:** 2021-12-17

**Authors:** Sarah Holmes, Aida Kuzucan, Abisola Olopoenia, Nicole Brandt, Becky Briesacher, Danya Qato, Barbara Zarowitz, Linda Wastila

**Affiliations:** 1 University of Maryland School of Nursing, Baltimore, Maryland, United States; 2 University of Maryland School of Pharmacy, Baltimore, Maryland, United States; 3 University of Maryland School of Pharmacy, University of Maryland School of Pharmacy, Maryland, United States; 4 Northeastern University, Boston, Massachusetts, United States

## Abstract

Nursing home residents are frequently prescribed antipsychotic and other psychopharmacologic medications (AP/PPM) to manage behavioral and psychological symptoms. Residents also experience care transitions between nursing homes and other healthcare institutions. Limited research exists on the relationship between AP/PPM use and care transitions in this population. The purpose of this study is to compare odds of care transitions among those with and without AP/PPM use, controlling for resident characteristics. This cross-sectional study used data from a 5% random sample of Medicare beneficiaries between 2011-2015 who resided in a nursing home and were continuously enrolled in Medicare Parts A, B, and D. Chi-square tests compared resident characteristics and AP/PPM use between those with <2 and ≥2 transitions. Multivariate logistic regression evaluated the odds of transitions in those with and without AP/PPM use during a one-year follow-up, including interaction effects among AP and PPM use. Among 132,263 eligible residents, the majority were female(68%), white(85%), and >75 years old(63%). Of residents who experienced ≥2 transitions, 17% received AP and 82% received PPM, compared to those with <2 transitions for which 9% and 66% received AP/PPM, respectively (p<.001). Logistic results showed that for residents with PPM use, those who take AP have 1.33 the odds of experiencing ≥2 transitions than PPM users who do not take AP (p<.001). Findings demonstrate a significant association between AP/PPM use and care transitions in nursing home residents. Prudent use of AP/PPM should thus be optimized with a goal of reducing care transitions and improving quality of care.

